# The structure and experience of interim roles in academic health sciences libraries

**DOI:** 10.5195/jmla.2025.1924

**Published:** 2025-04-18

**Authors:** John W. Cyrus, Roy E. Brown, Emily J. Hurst, Rasha Alsaadawi, Roy T. Sabo

**Affiliations:** 1 cyrusjw@vcu.edu, Research and Education Librarian, Health Sciences Library, Virginia Commonwealth University, Richmond, VA; 2 rebrown2@vcu.edu, Virginia Commonwealth University, Richmond, VA; 3 ehurst@hshsl.umaryland.edu, Health Sciences and Human Services Library, University of Maryland, Baltimore, MD; 4 alsaadawir@vcu.edu, Department of Biostatistics, School of Public Health, Virginia Commonwealth University, Richmond, VA; 5 rsabo@vcu.edu, Department of Biostatistics, School of Public Health, Virginia Commonwealth University, Richmond, VA

**Keywords:** Leadership, interim, Library management, Surveys and Questionnaire, academic health sciences libraries

## Abstract

**Objective:**

Interim leadership roles are commonly used in academic libraries to ensure continuity and oversight within the organization. Interim roles can be rewarding but fraught with challenges, including the assumption of responsibilities in unstable environments, unclear expectations, and poor organizational preparedness. This article presents findings from a survey of librarian's experiences serving in interim leadership positions.

**Methods:**

A survey was designed to capture perceptions of the structure of the leadership position and the experience of the interim leaders. It was distributed via social media and through health sciences library listservs. Responses were analyzed using descriptive statistics and exploratory one-way ANOVA to test for response differences between respondent sub-groups.

**Results:**

Fifty-four complete responses were collected. Respondents were predominantly White (89%) and female (77%). Seventy percent of respondents had worked in health sciences libraries for 11–25 years. Respondents indicated that expectations, expected duration, and transition plan for the role were unclear. Policies and procedures related to the interim role were lacking. Respondents agreed that full authority and acceptance were given as part of the role. There were statistically significant differences in responses relating to authority, retention, and acceptance by gender and race.

**Conclusions:**

Results show that interim leaders were given adequate authority and support, but that organizations were not necessarily prepared for the interim leader, lacking policies, procedures, and clear expectations related to the position. Libraries can better prepare for the future by creating permanent structures and policies to facilitate the transition into and out of interim leadership.

## OBJECTIVE

Interim leadership positions are one way for organizations to ensure continuity during staffing changes, recruitment, financial uncertainties, or other events beyond the control of the organization. While some interim leaders remain in their positions for a year or more, these positions are often intended to provide a temporary solution until a permanent manager or administrator is hired. Academic librarianship offers some insights into interim leadership, but there is limited health sciences library scholarship regarding how libraries and librarians can prepare for interim roles.

Existing literature on interim leadership in libraries includes personal narratives 1, 2], literature reviews [3], and, more recently, mixed methods studies of the experience of serving as an interim library leader 4, 5]. A consistent thread throughout the literature for both the organization and the individual is the importance of preparing for the interim position and setting shared expectations between the interim, the organization, and members of the organization 3–5].

Literature from other disciplines reveals a growing number of temporary leaders who serve in corporate settings, healthcare, and higher education. These temporary leaders often take on interim roles during times of transition, tension, or volatility, making their success in the position of particular importance to the organization [5]. Those selected for interim leadership positions come from both inside and outside of the organization. Those brought from the outside often have specialized skills and are usually brought in to not only bring about stability but also to address already identified areas of concern 6, 7]. Others are appointed from within because of their organizational knowledge and experience. While some individuals may be reluctant to take on an interim role, those that do may do so out of commitment to the organization 6, 8].

Interim leadership roles create significant opportunities for leadership development and maintaining continuity within health sciences libraries. However, interim positions may create additional uncertainty for the individual and organization if not planned appropriately, making it critical that individuals considering these opportunities carefully consider their career goals and the current state of their organization. Merritt, et al. suggest four points of consideration for individuals preparing to enter into an interim role: understanding expectations, adjusting expectations, accommodation (focus of pragmatic short-term action), and phasing out (setting the stage for a permanent leader) [9]. This proposed framework describes the cycle of work involved in a transitional leadership role, but does not articulate how the role may be shaped by organizational forces, such as planning and support.

In 2020, at a single institution, three librarians were each asked to serve in term-limited interim leadership positions at different levels of the organization. After completing these temporary assignments, the authors sought to better understand the landscape of interim roles in libraries and the expectations of interim leaders compared to their actual experiences. The goal of this research is to understand the perceptions and the experienced realities of those involved with interim leadership roles.

## METHODS

As there were no existing, validated instruments that captured all of the elements of interest to the researchers, the authors drew from existing research across multiple disciplines to create a survey specifically tailored to interim library leaders [3, 4, 10, 11]. The survey was composed of 27 questions on a 5-point Likert rating scale (strongly disagree=1 to strongly agree=5) and broken into three parts: structure of the interim position, experience in the interim position, and demographic information (see Supplemental File). This study was determined to be exempt from review by the Virginia Commonwealth University Institutional Review Board (HM20021475). The survey was created in QuestionPro [12] and distributed through listservs and through the social media platform Twitter, now X, in an attempt to solicit responses from those who held interim leadership positions in the last ten years. The survey was posted on Twitter using the library Twitter handle and through the following online mailing lists: Medlib-L, ACRL HSIG, and AAHSL-all. Because the exact number of health sciences librarians who have held interim leadership positions is not known, the researchers were unable to anticipate the exact number of responses that would be received.

Descriptive statistics were used to summarize the data from the responses. Complete responses were exported from the survey instrument into Microsoft Excel for analysis. Descriptive statistics (frequencies and percentages) were used to summarize survey respondents. Biostatisticians performed exploratory analysis using one-way analysis of variance (ANOVA) models to investigate associations between individual experience in the interim position and each of the following (separately) as predictors: (i) the level of the interim position, (ii) time in the library profession, and (iii) the race and gender of the participant. A one-way ANOVA model was also used to investigate the association between the structure of the interim position and the type of institution. While broader data on demographics were collected, demographic variables were grouped as follows for analysis: sex (male and female), race (white or Caucasian, other, and missing), level of position (department head/assistant or deputy director and director or dean), years in the profession (0–10, 11–20, 21–30, 31+), institution type (academic health sciences, college/university, and other). All summaries and statistical analyses were computed in the R statistical software [13].

## RESULTS

The survey received 54 complete responses. Demographic summaries of the respondents can be found in Table 1. The majority of respondents were white (89%), female (77%), and working in health sciences libraries with 25 or fewer full-time employees (74%). Seventy percent (70%) of respondents reported 11–25 years of experience in libraries. Most held interim positions at the Director level (61%) while more than a quarter held interim positions at the Department Head level (26%). In addition to demographic information, participants were asked to respond to two sets of statements: one describing aspects of the structure of the interim position and the other describing their experience in the interim position.

**Table 1:** Demographic characteristics of respondents for groups for which there were responses. A full list of the demographic variables collected by the survey can be seen in the Supplementary Material.

Demographic Variable	Number of Responses (n=54)	%
Institution Type		
Academic health sciences	38	70.37%
College/University	13	24.07%
Hospital	1	1.85%
Medical or health sciences association or society (non-profit)	1	1.85%
Research or health research center	1	1.85%
Library FTEs		
1–5	10	18.52%
6–10	9	16.67%
11–25	21	38.89%
26–30	5	9.26%
31–35	3	5.56%
36–40	2	3.70%
41–45	1	1.85%
46–50	2	3.70%
More than 50 FTE	1	1.85%
Level of Interim Position		
Department Head	14	25.93%
Associate or Deputy Director	5	9.26%
Director	33	61.11%
Other	2	3.70%
Gender Identity		
Female	44	77.19%
Male	10	17.54%
Cisgender	3	5.26%
Racial or Ethnic Group		
American Indian or Alaska Native	1	1.82%
Asian	3	5.45%
Black or African-American	1	1.82%
Hispanic, Latino, or Spanish Origin	1	1.82%
White or Caucasian	49	89.09%
Years of Library Experience		
0–5 years	2	3.70%
6–10 years	2	3.70%
11–15 years	15	27.78%
16–20 years	10	18.52%
21–25 years	13	24.07%
26–30 years	7	12.96%
31 or more years	5	9.26%
Years at Present Institution		
0–5 years	8	14.81%
6–10 years	16	29.63%
11–15 years	10	18.52%
16–20 years	11	20.37%
21–25 years	4	7.41%
26–30 years	4	7.41%
31 or more years	1	1.85%

### Structure of Interim Position

**Figure 1:**
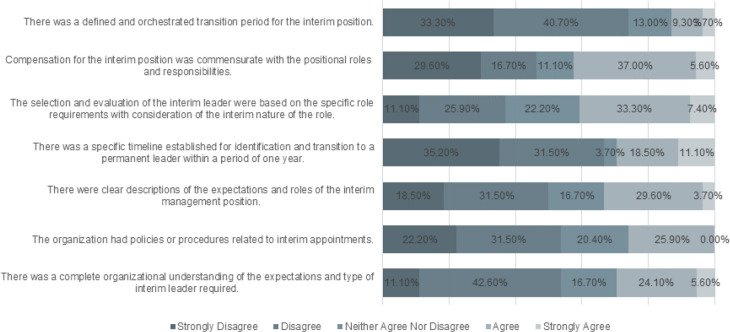
Responses to Questions on the Structure of the Interim Position

Questions in this section of the survey asked respondents to indicate their agreement regarding organizational preparedness and existing structures put into place for an interim leadership position (Figure 1). Most respondents disagreed (disagree and strongly disagree) with the statement that there was an understanding of the expectations of the interim position or duties anticipated by the organization during the interim period (53.7%). Similarly, most respondents disagreed that their organization had policies and procedures in place related to interim appointments (53.7%).

Opinions among respondents were slightly divided regarding whether or not the expectations of the interim role were clearly described for their organization with half of the respondents disagreeing or strongly disagreeing (n=27, 50%) and one-third agreeing or strongly agreeing (n=18, 33.3%). A greater degree of disagreement was observed among respondents regarding whether a timeline for transitioning from an interim to permanent was established with two-thirds of respondents disagreeing or strongly disagreeing with the statement (n=36, 66.7%). Respondents were neutral on the issue of whether the interim leader was selected and evaluated based on the role requirements with nearly one quarter of respondents neither agreeing nor disagreeing (n=12, 22.2%). Responses were divided on whether compensation for the interim position was commensurate with the positional roles and responsibilities with 46.3% (n=25) disagreeing or strongly disagreeing and 42.6% (n=23) agreeing or strongly agreeing.

The majority of respondents felt that transitioning into and out of the interim position was not well planned with 74% (n=40) disagreeing or strongly disagreeing that there was a defined transition period for the interim position. The ANOVA results indicated no significant differences in responses across institution types for any statement related to the structure of the interim position

### Experience in the Interim Position

**Figure 2:**
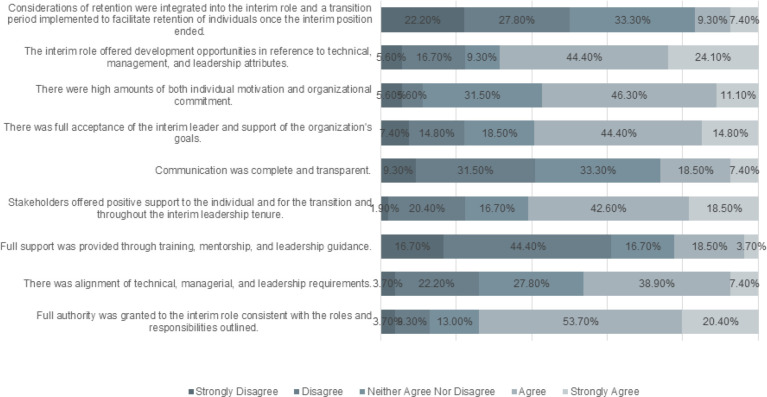
Responses to Questions on Experiences in the Interim Position

Questions in this section of the survey asked respondents to indicate their level of agreement regarding their firsthand experience in the interim leadership position (Figure 2). Most respondents agreed that the interim position was granted full authority based on the understood roles and responsibilities (n=40, 74.1%). There was a moderate amount of agreement regarding whether there was alignment between the technical, managerial, and leadership requirements required in the position with 46.3% either agreeing or strongly agreeing (n=25).

More than half of the respondents (n=33, 61.1%) disagreed that they received support in the form of training, mentorship, or leadership guidance while in the role. However, many respondents (n=33, 61.1%) indicated that they received positive support from stakeholders while in the interim position. Respondents were more divided on whether or not communication was complete and transparent during the interim position with 40.8% disagreeing and one third (33.3%) neither agreeing nor disagreeing.

Many respondents (59.2%) agreed that there was full acceptance of the interim leader and the organization's goals for this position, and more than half of respondents (57.4%) agreed that levels of individual motivation and organizational commitment were high during the interim period. Additionally, the majority of respondents (68.5%) agreed that the interim role offered opportunities for the development of managerial and leadership skills. Despite this, 50.0% of respondents disagreed that considerations of retention of the interim leader were integrated into the role including a period of transition out of the role.

ANOVA testing (see Table 2) revealed no significant differences in responses on experience in the interim role by the level of the interim position or the time in the profession. A statistically significant difference in response rates was found between male and female for the statement on whether full authority was granted to the interim role (p = 0.0284). Males (mean (SE)=4.40 (0.31)) were more likely than females (3.64 (0.15)) to report that full authority was granted in the interim position (p-value = 0.0284). Significant differences in responses were also observed by race on the statements relating to whether considerations of retention were integrated into the interim role (p = 0.0305), where White race (2.44 (0.16)) were less likely than other races (3.60 (0.50)) to agree with statements relating to considerations of retention after the interim positions (p-value = 0.0305). Also, White race (3.38 (0.15)) were less likely than other races (4.60 (0.47)) to agree that there was full acceptance of the interim leader and support of the organization's goals. (p-value = 0.0166). Significant results of the analysis are presented in Table 2 with the remaining analyses available in the Supplementary File.

**Table 2:** ANOVA Model Results for Significant Differences

Parameter	DF	Sum Sq	Mean Sq	F Value	P-Value
Full authority was granted to the interim role consistent with the roles and responsibilities outlined.					
Gender	1	4.75	4.75	5.09	0.0284^*^
Residuals	52	48.58	0.93		
Considerations of retention were integrated into the interim role and a transition period was implemented to facilitate retention of individuals once the interim position ended.					
Race	1	6.12	6.12	4.95	0.0305^*^
Residuals	51	63.01	1.24		
There was full acceptance of the interim leader and support of the organization's goals.					
Race	1	6.80	6.80	6.14	0.0166*
Residuals	51	56.45	1.11		

### Other Findings

The survey also asked several questions outside of either of the two domains or demographics to better understand factors that may have contributed to the experience of an interim leader. For instance, more than one third of respondents (n = 19; 35%) indicated that they were still in an interim role at the time of the survey. Based on data provided by 17 of these 19 respondents (two respondents did not provide an interim appointment start date), the average time in an interim role was 11.5 months, but the length of the appointment lasted anywhere from three to thirty months.

A majority of participants (74.07%; n=40) reported that they maintained all typical responsibilities in addition to taking on the responsibilities of the interim position while 20% (n=11) reported maintaining only limited responsibilities from their previous position. Additionally, 91% (n=49) of respondents indicated that they had taken part in a formal leadership training program before to taking on the interim position. A breakdown of common programs shows that: ten had participated in the National Library of Medicine (NLM)/Association of Academic Health Sciences Libraries (AAHSL) Leadership Fellows Program, nine took part in an institutional leadership program, and eight took part in the Harvard Leadership Institute for Academic Librarians while 19 had taken part in another form of training or program not listed in the survey.

## DISCUSSION

In this survey of interim leaders in health sciences libraries, participants indicated that the experience was a good opportunity for professional development and they were largely given adequate authority and support to fulfill their responsibilities. However, responses to questions on authority, retention, and acceptance exhibited statistically significant differences by gender (authority) and race (retention and acceptance). Respondents also indicated that organizations were not necessarily prepared for the interim leader, lacking policies and procedures or clear expectations related to the duration of the interim position, retention of existing duties, and transitioning from an interim to a permanent leader.

This survey found a significant difference between male and female participants' perceptions of whether full authority consistent with the roles and responsibilities of the interim position was granted. The perception of authority granted a position may vary based on several factors. Gender stereotypes play an important role, as they influence how others perceive a leader regardless of their performance. Due to historic underrepresentation within library leadership roles, women are not always viewed as leaders by their colleagues, resulting in actual or perceived reduction of authority [14]. Additionally, research indicates that women in leadership roles are conscious of the risk of not being accepted by their subordinates, which can lead to lower expectations of influence and more negative self-evaluation of leadership opportunities [15].

At the same time, research shows that stereotypes of leadership within primarily female organizations tends to be more feminine, and that female dominated organizations tend to view female leaders as more effective [16,17]. The contrast between the context and the experience of interim leaders in health sciences libraries suggests that other factors may dictate the real or perceived authority granted to interim leaders.

We also found that participants of White race were less likely to agree that considerations of retention were made after the interim period ended. This is an unexpected finding that warrants further exploration. However, there is research that may offer some explanation of this finding. The period immediately following an interim appointment can be difficult for those leaving the role, evoking strong emotional responses ranging from relief and happiness to disappointment or bitterness [18]. For interim leaders that do who not immediately depart the institution or are promoted to a permanent role, remaining at the institution or reverting to a prior role can be challenging. Returning to a prior role with the same responsibilities can be a very uncertain time as the demands and social context have changed [4]. Research on morale and retention in academic libraries has found that turnover is partly linked to dissatisfaction based on the perceived potential for advancement or promotion (to a higher position, not a higher academic rank). Librarians reported that staying at the same institution meant forgoing opportunities and suffering from a lack of opportunities to acquire the skills and experience needed for a more advanced position [19]. These factors, uncertainty of returning to a prior role and perception of low potential for advancement, may dictate the perception of efforts to retain interim leaders and may lead to a situation of involuntary staying-a state where, after a trigger event, an employee is forced to reevaluate their understanding of the organization and their role within it [20].

Our analysis also indicated that participants of White race were less likely than other races to agree that there was full acceptance of the interim leader. Gaining acceptance is an important goal for an interim leader and the organization and should be a key aim of the onboarding process. This socialization helps to integrate the interim leader into the organization by equipping them with the necessary knowledge and skills to exercise the authority¬ they are given [21]. Regardless of socialization, which may be minimized in some libraries based on the responses to questions in this survey about the structure of interim positions, factors like gender and race can impact acceptance. The perception of leadership efficacy varies by race and is affected by social and organizational structures that prioritize whiteness [22]. Given these findings, we might expect the results of this survey to indicate that races other than White were less likely to agree that there was full acceptance of the interim position. Our findings show the opposite may indicate that there are other contextual or organizational factors at work.

The lack of structure and policies related to interim positions in health sciences libraries may stem from many sources, including the relative frequency of requiring an interim leader or from the circumstances under which an interim leader is required. When a transition in leadership is anticipated, for instance due to retirement or planned personal leave, there may be sufficient time to fully consider the requirements for an interim position. Unexpected conditions necessitating interim leadership, such as unanticipated health concerns or layoffs, may present greater challenges for the organization and result in less planning for the position [5].

Management literature suggests several potential solutions to the problem of creating a plan for interim roles before they are required. One such solution would be to describe and implement a cycle for interim assignments tailored to the needs of the organization. The cycle of Preparation (determining scope and learning about culture), Entry (establishing authority and priorities), Delivery (completing the objective of the assignment), and Exit (knowledge transfer to permanent leaders) described by Woods provides a useful framework for thinking about interim responsibilities and establishing appropriate resources and support for the position at the organization level [11]. Considering each stage in the cycle could also help alleviate issues related to transitions, specifically how the interim leader transitions out of the role, whether or not they transition back to a prior position, or whether or not they are retained at all. Each of these results has specific implications that need to be considered [23]. Parchment, et al. suggest a similar approach based in the creation of a formal manual outlining a framework for progressing in an interim role from a transition phase to applying and expanding on leadership skills [24]. This model, based on the American Organization for Nursing Leadership competencies, provides detailed suggestions for the topics covered at each phase (Initial Transition, Reality, Accommodation); however, while an exit strategy is mentioned, little time is spent describing the important step of transitioning out of the interim role can be accomplished.

Aside from a deficit in planning for interim positions through policy, responses to the survey indicated that organizations did not appear to be clear on specific skills required of the interim manager for the role. This includes planned development for the interim while in the position. London proposes a number of considerations for interim assignments, including power dynamics and skill development while in the interim role, addressing the respective roles of the organization, senior leadership, and the interim in planning for and navigating these complexities [25]. Other research emphasizes the importance of mentorship throughout the interim period. Mentoring in this case includes receiving assistance navigating new power dynamics as an organizational leader and complex relationships between former peers and a temporary position as a manager or supervisor [26,27]. Providing both general orientations as well as tailored development may be an important way that health sciences libraries can strengthen support for interim leadership.

There are some limitations to these findings that need to be considered. The survey instrument developed for this project was based on research in another discipline, which could create misalignment between the core concepts driving the questions and the perception of respondents. Additionally, the instrument was not validated during development. This survey was conducted during the Covid-19 pandemic, which could have exacerbated unorthodox conditions in which interim leaders were brought into organizations. The respondents to the survey were 89% Caucasian/White and 77% female. A recent survey conducted with Medical Library Association (MLA) members found the organization to be 73% white and 79% female [28]. A similar racial/ethnic distribution was observed in a cross-sectional analysis of interim deans at medical schools in the United States [29]. Based on this data, our sample may overrepresent white librarians who have served in interim leadership roles or indicate a disparity in opportunities for librarians of other races and ethnicities to take on these positions. Lastly, the research team was comprised of two white cis gender males and one white cis gender female librarians indicating that survey questions and structure may be limited based on the privilege and lived experiences of the research team.

Overall, these results showed that participants viewed their experiences as interim leaders as providing many positive experiences. At the same time, libraries and library leadership have work to do in terms of developing permanent structures and policies to facilitate the transition into and out of interim leadership roles. Further research should be conducted to understand how the implementation of specific planning impacts the individual and organizational experience of transitional leaders. Additionally, creating an environment that grants all leaders, regardless of race or gender, the same level of authority and acceptance is critically important for health sciences librarianship. Future research should explore the relationships revealed here between the experiences of male and female participants and between the experiences of participants of different races.

## Data Availability

Deidentified data associated with this is publicly available via the Open Science Framework project associated with this work (DOI 10.17605/OSF.IO/4HDXY).

## SUPPLEMENTAL FILES

**Appendix A** : Full Statistical Analysis
