# Supplementary material for: The structure and experience of interim roles in academic health sciences libraries
Source: J Med Libr Assoc. 2025 Apr 18;113(2):148–57. doi: 10.5195/jmla.2025.1924 (PMC12058337; doi:10.5195/jmla.2025.1924)
Supplement: Supplementary file 1 — Appendix A : Full Statistical Analysis [file jmla-113-2-148-s01.docx]

**Supplementary File – Full ANOVA Results**

Table 1: Summary of Characteristics of participants used in ANOVA

| **Characteristic** | **Category** | **Frequency (%)** |
| --- | --- | --- |
| Sex | Female | 44 (82%) |
|  | Male | 10 (18%) |
| Race | Other | 5 (9%) |
|  | White or Caucasian | 48 (89%) |
|  | Missing | 1 (2%) |
| level of your interim position | Department Head or Associate or Deputy Director | 19 (35%) |
|  | Director or Dean | 35 (65%) |
| Time in the library profession | 0-10 years | 4 (8%) |
|  | 11-20 years | 25 (46%) |
|  | 21-30 years | 20 (37%) |
|  | 31 or more years | 5 (9%) |
| Primary Institution Type | Academic health sciences | 38 (70%) |
|  | College/University | 13 (24%) |
|  | Other | 3 (6%) |

One-way analysis of variance (ANOVA) models are used to investigate associations between individual experience in the interim position (as the outcome) and the level of the interim position as predictor. The individual experience in the interim position is represented by the Likert score (from strongly disagree=1, to strongly agree=5) for six statements:

Table 2: ANOVA Model Results for Stakeholders offered positive support to the individual and for the transition and throughout the interim leadership tenure.

| Parameter | DF | Sum Sq | Mean Sq | F Value | P-Value |
| --- | --- | --- | --- | --- | --- |
| Interim position level | 1 | 0.03 | 0.03 | 0.02 | 0.8846 |
| Residuals | 52 | 61.31 | 1.18 |  |  |

Table 3: ANOVA Model Results for The interim role offered development opportunities in reference to technical, management, and leadership attributes.

| Parameter | DF | Sum Sq | Mean Sq | F Value | P-Value |
| --- | --- | --- | --- | --- | --- |
| Interim position level | 1 | 0.23 | 0.23 | 0.16 | 0.6891 |
| Residuals | 52 | 74.08 | 1.42 |  |  |

Table 4: ANOVA Model Results for Considerations of retention were integrated into the interim role and a transition period implemented to facilitate retention of individuals once the interim position ended.

| Parameter | DF | Sum Sq | Mean Sq | F Value | P-Value |
| --- | --- | --- | --- | --- | --- |
| Interim position level | 1 | 0.11 | 0.11 | 0.08 | 0.7812 |
| Residuals | 52 | 71.37 | 1.37 |  |  |

Table 5: ANOVA Model Results for There was full acceptance of the interim leader and support of the organization's goals.

| Parameter | DF | Sum Sq | Mean Sq | F Value | P-Value |
| --- | --- | --- | --- | --- | --- |
| Interim position level | 1 | 0.26 | 0.26 | 0.25 | 0.6182 |
| Residuals | 52 | 53.08 | 1.02 |  |  |

Table 6: ANOVA Model Results for There was full acceptance of the interim leader and support of the organization's goals.

| Parameter | DF | Sum Sq | Mean Sq | F Value | P-Value |
| --- | --- | --- | --- | --- | --- |
| Interim position level | 1 | 0.002 | 0.002 | 0.002 | 0.9697 |
| Residuals | 52 | 63.48 | 1.22 |  |  |

Table 7: ANOVA Model Results for There was full acceptance of the interim leader and support of the organization's goals.

| Parameter | DF | Sum Sq | Mean Sq | F Value | P-Value |
| --- | --- | --- | --- | --- | --- |
| Interim position level | 1 | 0.20 | 0.20 | 0.15 | 0.7022 |
| Residuals | 52 | 69.14 | 1.33 |  |  |

One-way analysis of variance (ANOVA) models are used to investigate associations between individual experience in the interim position (as the outcome) and time in the library as predictor. The individual experience in the interim position is represented by the Likert score (from strongly disagree=1, to strongly agree=5) for six statements:

Table 8: ANOVA Model Results for Stakeholders offered positive support to the individual and for the transition and throughout the interim leadership tenure.

| Parameter | DF | Sum Sq | Mean Sq | F Value | P-Value |
| --- | --- | --- | --- | --- | --- |
| Time in profession | 3 | 0.82 | 0.27 | 0.23 | 0.8773 |
| Residuals | 50 | 60.51 | 1.21 |  |  |

Table 9: ANOVA Model Results for The interim role offered development opportunities in reference to technical, management, and leadership attributes.

| Parameter | DF | Sum Sq | Mean Sq | F Value | P-Value |
| --- | --- | --- | --- | --- | --- |
| Time in profession | 3 | 2.38 | 0.79 | 0.55 | 0.6504 |
| Residuals | 50 | 71.94 | 1.44 |  |  |

Table 10: ANOVA Model Results for Considerations of retention were integrated into the interim role and a transition period implemented to facilitate retention of individuals once the interim position ended.

| Parameter | DF | Sum Sq | Mean Sq | F Value | P-Value |
| --- | --- | --- | --- | --- | --- |
| Time in profession | 3 | 1.92 | 0.64 | 0.46 | 0.7112 |
| Residuals | 50 | 69.56 | 1.39 |  |  |

Table 11: ANOVA Model Results for There was full acceptance of the interim leader and support of the organization's goals.

| Parameter | DF | Sum Sq | Mean Sq | F Value | P-Value |
| --- | --- | --- | --- | --- | --- |
| Time in profession | 3 | 0.59 | 0.20 | 0.19 | 0.9044 |
| Residuals | 50 | 52.74 | 1.05 |  |  |

Table 12: ANOVA Model Results for There was full acceptance of the interim leader and support of the organization's goals.

| Parameter | DF | Sum Sq | Mean Sq | F Value | P-Value |
| --- | --- | --- | --- | --- | --- |
| Time in profession | 3 | 2.74 | 0.91 | 0.75 | 0.5262 |
| Residuals | 50 | 60.74 | 1.21 |  |  |

Table 13: ANOVA Model Results for There was full acceptance of the interim leader and support of the organization's goals.

| Parameter | DF | Sum Sq | Mean Sq | F Value | P-Value |
| --- | --- | --- | --- | --- | --- |
| Time in profession | 3 | 1.98 | 0.66 | 0.49 | 0.6903 |
| Residuals | 50 | 67.35 | 1.35 |  |  |

One-way analysis of variance (ANOVA) models are used to investigate associations between individual experience in the interim position (as the outcome) and gender of the participant as predictor. The individual experience in the interim position is represented by the Likert score (from strongly disagree=1, to strongly agree=5) for six statements:

Table 14: ANOVA Model Results for Stakeholders offered positive support to the individual and for the transition and throughout the interim leadership tenure.

| Parameter | DF | Sum Sq | Mean Sq | F Value | P-Value |
| --- | --- | --- | --- | --- | --- |
| Gender | 1 | 2.42 | 2.42 | 2.14 | 0.1495 |
| Residuals | 52 | 58.91 | 1.13 |  |  |

Table 15: ANOVA Model Results for The interim role offered development opportunities in reference to technical, management, and leadership attributes.

| Parameter | DF | Sum Sq | Mean Sq | F Value | P-Value |
| --- | --- | --- | --- | --- | --- |
| Gender | 1 | 2.51 | 2.51 | 1.81 | 0.1838 |
| Residuals | 52 | 71.81 | 1.38 |  |  |

Table 16: ANOVA Model Results for Considerations of retention were integrated into the interim role and a transition period implemented to facilitate retention of individuals once the interim position ended.

| Parameter | DF | Sum Sq | Mean Sq | F Value | P-Value |
| --- | --- | --- | --- | --- | --- |
| Gender | 1 | 0.08 | 0.08 | 0.06 | 0.8085 |
| Residuals | 52 | 71.40 | 1.37 |  |  |

Table 17: ANOVA Model Results for There was full acceptance of the interim leader and support of the organization's goals.

| Parameter | DF | Sum Sq | Mean Sq | F Value | P-Value |
| --- | --- | --- | --- | --- | --- |
| Gender | 1 | 4.75 | 4.75 | 5.09 | 0.0284* |
| Residuals | 52 | 48.58 | 0.93 |  |  |

Table 18: ANOVA Model Results for There was full acceptance of the interim leader and support of the organization's goals.

| Parameter | DF | Sum Sq | Mean Sq | F Value | P-Value |
| --- | --- | --- | --- | --- | --- |
| Gender | 1 | 1.25 | 1.25 | 1.04 | 0.3125 |
| Residuals | 52 | 62.24 | 1.20 |  |  |

Table 19: ANOVA Model Results for There was full acceptance of the interim leader and support of the organization's goals.

| Parameter | DF | Sum Sq | Mean Sq | F Value | P-Value |
| --- | --- | --- | --- | --- | --- |
| Gender | 1 | 0.80 | 0.80 | 0.61 | 0.4390 |
| Residuals | 52 | 68.53 | 1.32 |  |  |

One-way analysis of variance (ANOVA) models are used to investigate associations between individual experience in the interim position (as the outcome) and race of the participant as predictor. The individual experience in the interim position is represented by the Likert score (from strongly disagree=1, to strongly agree=5) for six statements:

Table 20: ANOVA Model Results for Stakeholders offered positive support to the individual and for the transition and throughout the interim leadership tenure.

| Parameter | DF | Sum Sq | Mean Sq | F Value | P-Value |
| --- | --- | --- | --- | --- | --- |
| Race | 1 | 1.04 | 1.04 | 0.88 | 0.3515 |
| Residuals | 51 | 59.98 | 1.18 |  |  |

Table 21: ANOVA Model Results for The interim role offered development opportunities in reference to technical, management, and leadership attributes.

| Parameter | DF | Sum Sq | Mean Sq | F Value | P-Value |
| --- | --- | --- | --- | --- | --- |
| Race | 1 | 0.08 | 0.08 | 0.06 | 0.8115 |
| Residuals | 51 | 71.47 | 1.40 |  |  |

Table 22: ANOVA Model Results for Considerations of retention were integrated into the interim role and a transition period implemented to facilitate retention of individuals once the interim position ended.

| Parameter | DF | Sum Sq | Mean Sq | F Value | P-Value |
| --- | --- | --- | --- | --- | --- |
| Race | 1 | 6.12 | 6.12 | 4.95 | 0.0305* |
| Residuals | 51 | 63.01 | 1.24 |  |  |

Table 23: ANOVA Model Results for Full authority was granted to the interim role consistent with the roles and responsibilities outlined.

| Parameter | DF | Sum Sq | Mean Sq | F Value | P-Value |
| --- | --- | --- | --- | --- | --- |
| Race | 1 | 1.10 | 1.09 | 1.10 | 0.2990 |
| Residuals | 51 | 50.72 | 0.99 |  |  |

Table 24: ANOVA Model Results for Full support was provided through training, mentorship, and leadership guidance.

| Parameter | DF | Sum Sq | Mean Sq | F Value | P-Value |
| --- | --- | --- | --- | --- | --- |
| Race | 1 | 4.57 | 4.57 | 3.97 | 0.0517 |
| Residuals | 51 | 58.68 | 1.15 |  |  |

Table 25: ANOVA Model Results for There was full acceptance of the interim leader and support of the organization's goals.

| Parameter | DF | Sum Sq | Mean Sq | F Value | P-Value |
| --- | --- | --- | --- | --- | --- |
| Race | 1 | 6.80 | 6.80 | 6.14 | 0.0166* |
| Residuals | 51 | 56.45 | 1.11 |  |  |

One-way ANOVA models are used to investigate the association between the structure of the interim position (as the outcome) and the type of institution (as a predictor). The structure of the interim position is represented by the Likert score (from strongly disagree=1, to strongly agree=5)

Table 26: ANOVA Model Results for The organization had policies or procedures related to interim appointments.

| Parameter | DF | Sum Sq | Mean Sq | F Value | P-Value |
| --- | --- | --- | --- | --- | --- |
| Primary institution type | 2 | 2.56 | 1.28 | 1.04 | 0.3612 |
| Residuals | 51 | 62.94 | 1.23 |  |  |

Table 27: ANOVA Model Results for There were clear descriptions of the expectations and roles of the interim management position.

| Parameter | DF | Sum Sq | Mean Sq | F Value | P-Value |
| --- | --- | --- | --- | --- | --- |
| Primary institution type | 2 | 1.22 | 0.61 | 0.42 | 0.6614 |
| Residuals | 51 | 74.43 | 1.46 |  |  |

Table 28: ANOVA Model Results for There was a specific timeline established for identification and transition to a permanent leader within a period of one year.

| Parameter | DF | Sum Sq | Mean Sq | F Value | P-Value |
| --- | --- | --- | --- | --- | --- |
| Primary institution type | 2 | 0.38 | 0.19 | 0.09 | 0.9124 |
| Residuals | 51 | 106.45 | 2.09 |  |  |

Table 29: ANOVA Model Results for The selection and evaluation of the interim leader were based on the specific role requirements with consideration of the interim nature of the role.

| Parameter | DF | Sum Sq | Mean Sq | F Value | P-Value |
| --- | --- | --- | --- | --- | --- |
| Primary institution type | 2 | 3.72 | 1.86 | 1.39 | 0.2589 |
| Residuals | 51 | 68.28 | 1.34 |  |  |

Table 30: ANOVA Model Results for There was a defined and orchestrated transition period for the interim position.

| Parameter | DF | Sum Sq | Mean Sq | F Value | P-Value |
| --- | --- | --- | --- | --- | --- |
| Primary institution type | 2 | 0.60 | 0.30 | 0.25 | 0.7823 |
| Residuals | 51 | 61.94 | 1.21 |  |  |
